# Sex-type specification and meristem development in fern gametophytes

**DOI:** 10.1007/s00427-025-00728-6

**Published:** 2025-11-10

**Authors:** Dinh Nhan Lai, Yun Zhou

**Affiliations:** 1 Department of Botany and Plant Pathology, Purdue University, West Lafayette, IN 47907 USA; 2 Purdue Center for Plant Biology, Purdue University, West Lafayette, IN 47907 USA

## Abstract

Sex determination, involving the physiological and morphological separation of male and female gamete-producing structures, promotes outcrossing and helps maintain genetic diversity in land plants. In homosporous ferns, which lack sex chromosomes, sex determination is specified during the gametophyte stage and is regulated by intergametophytic signaling mediated by the pheromone antheridiogen. *Ceratopteris richardii* has long served as a model for dissecting the molecular and cellular mechanisms underlying fern sex determination and associated meristem development in fern gametophytes, and in recent years, important new progress has been achieved. In this review, we summarize recent findings that uncover key environmental and molecular factors influencing antheridiogen responses and highlight the cellular basis of sex-type specification and the initiation and maintenance of meristems in *Ceratopteris* gametophytes. We also discuss emerging directions in the field, emphasizing the importance of spatial expression dynamics and interacting regulatory networks in fern sexual differentiation. Future advances will likely be driven by integrating genetic tools, multi-omics platforms, and quantitative live-imaging approaches, offering a more comprehensive view of the regulatory logic underlying sex determination and meristem development in ferns.

## Introduction

Sex determination, involving the physiological and morphological separation of male and female gamete-producing structures, is a fundamental process for sexual reproduction and species persistence in land plants [1]. Unlike many other multicellular organisms, homosporous ferns do not possess sex chromosomes. Instead, their sex types are specified during their gametophyte phase, producing meristem-containing hermaphroditic (or female) gametophytes and ameristic male gametophytes [1]. Hermaphrodites carry both female and male gametangia and can self-fertilize to generate homozygous sporophytes. However, this inbreeding strategy potentially leads to the accumulation of deleterious alleles and reduced genetic variation, thereby lowering population fitness. Indeed, previous studies show that, despite the presence of hermaphrodites, most wild ferns tend to reproduce by outcrossing [2]. To promote outcrossing, ferns have evolved a pheromone, antheridiogen-based signaling system within the population, which directs sex expression and adjusts population sex ratios [3–6]. This system has been demonstrated to be employed in sex determination among diverse fern species across different taxa [7, 8], and recent field evidence confirms its role in regulating sex ratios under natural conditions [9].

Among homosporous fern species, *Ceratopteris richardii* (hereafter *Ceratopteris*) has become a widely used model organism to address various fundamental developmental processes, including the molecular and cellular mechanisms of sex determination and associated meristem development [6, 8, 10–22]. This review summarizes recent advances in understanding sex determination and sex-type switching in *Ceratopteris* gametophytes, along with the associated processes of meristem initiation and maintenance. We highlight key environmental and genetic factors that influence antheridiogen responses and sex-type specification, discuss recent tool development, and outline future research directions in this rapidly advancing field.

## From genetically identical spores to male or hermaphroditic gametophytes

**Fig. 1:**
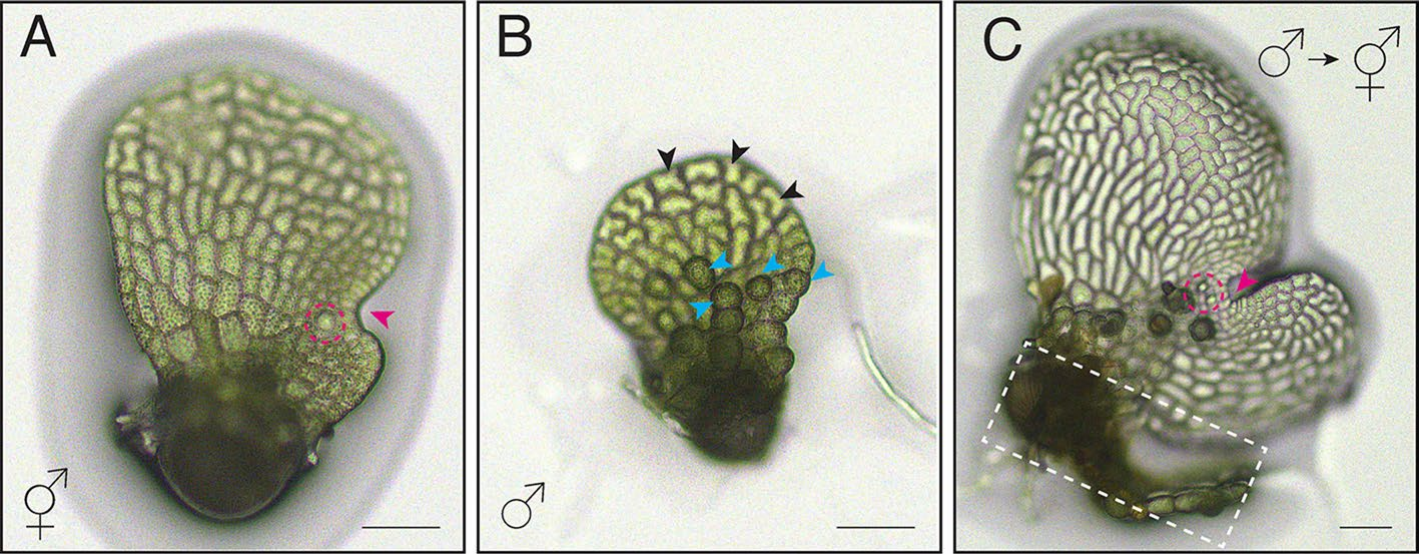
Sex types in *Ceratopteris* Gametophytes. (**A**-**C**) Light micrographs showing a *Ceratopteris* hermaphrodite (**A**), a male (**B**), and male-to-hermaphrodite conversion (**C**). Magenta arrowheads: meristem in (**A**) and *de novo* meristem in (**C**); magenta dashed circles in (**A**, **C**): archegonia; blue arrowheads in (**B**): representative antheridia; black arrowheads in (**B**): representative non-antheridium cells; white-dashed box in (**C**): original male body from which the *de novo* meristem forms. Scale bars in (**A**-**C**): 100 μm

**Fig. 2:**
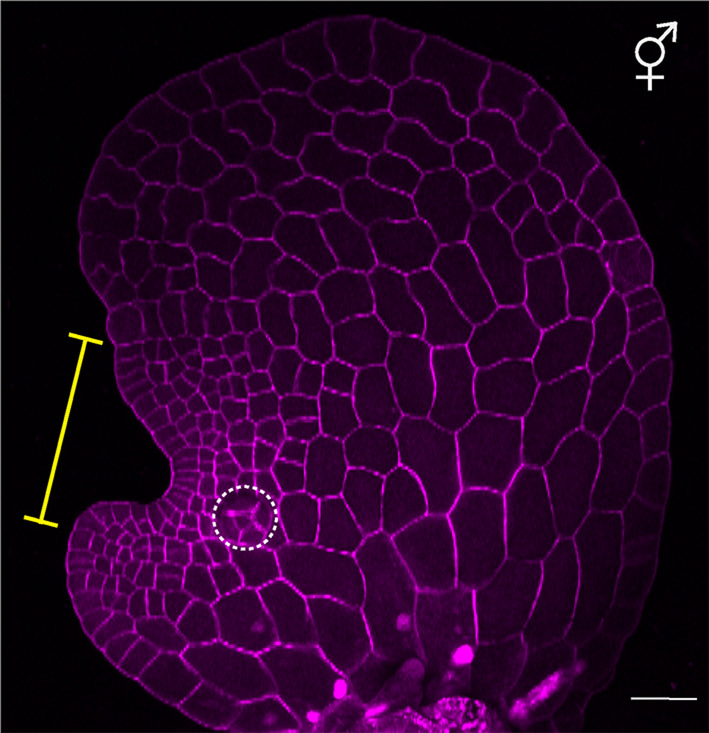
The multicellular meristem and adjacent archegonia in *Ceratopteris* hermaphrodites. A hermaphrodite (5 days after germination) stained and imaged by confocal microscopy. The hermaphrodite develops a multicellular meristem on one lateral side of the prothallus, characterized by small cells forming a concave notch. Yellow bar: meristem notch region; white dash circle: developing archegonium adjacent to the meristem. Magenta: cell outlines. Scale bar: 50 μm

**Fig. 3:**
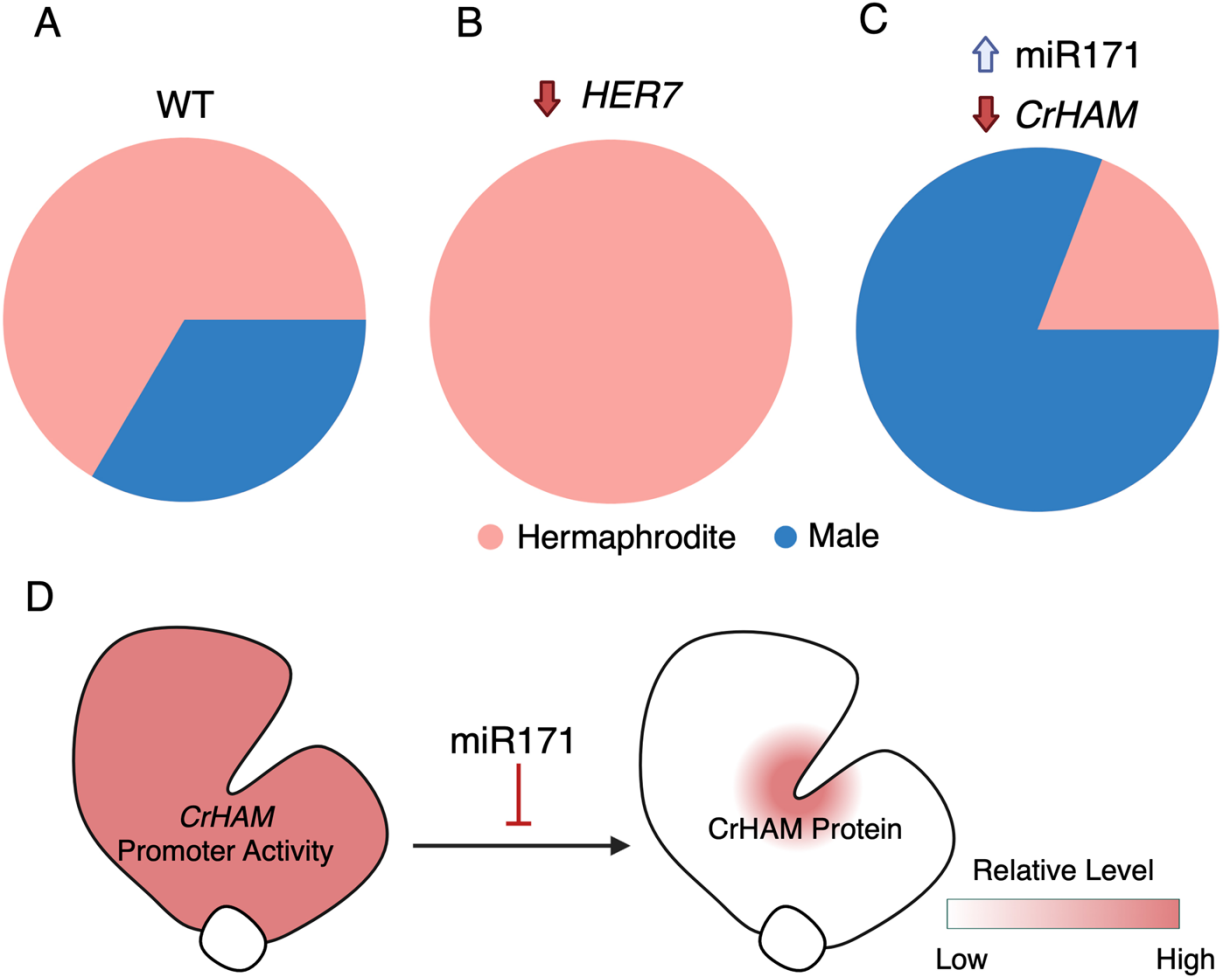
Sex-type specification pathways in *Ceratopteris* gametophytes. (**A**-**C**) Pie charts illustrating sex ratios in *Ceratopteris* gametophyte populations of different genetic backgrounds, indicating the proportion of hermaphrodites (pink) and males (blue). (**A**) *Ceratopteris* wild type (WT). (**B**) *her7* mutants (adapted from Banks, 1994) (**C**) *CrHAM* loss-of-function and *miR171* gain-of-function lines (adapted from Geng et al., 2024). (**D**) Diagram illustration of the miR171-CrHAM regulatory module in *Ceratopteris* gametophytes. *CrHAM* promoter activity and CrHAM protein accumulation are illustrated on a scale from white (none) to red (high). The figure was created using BioRender and further modified in PowerPoint for clarity

**Fig. 4:**
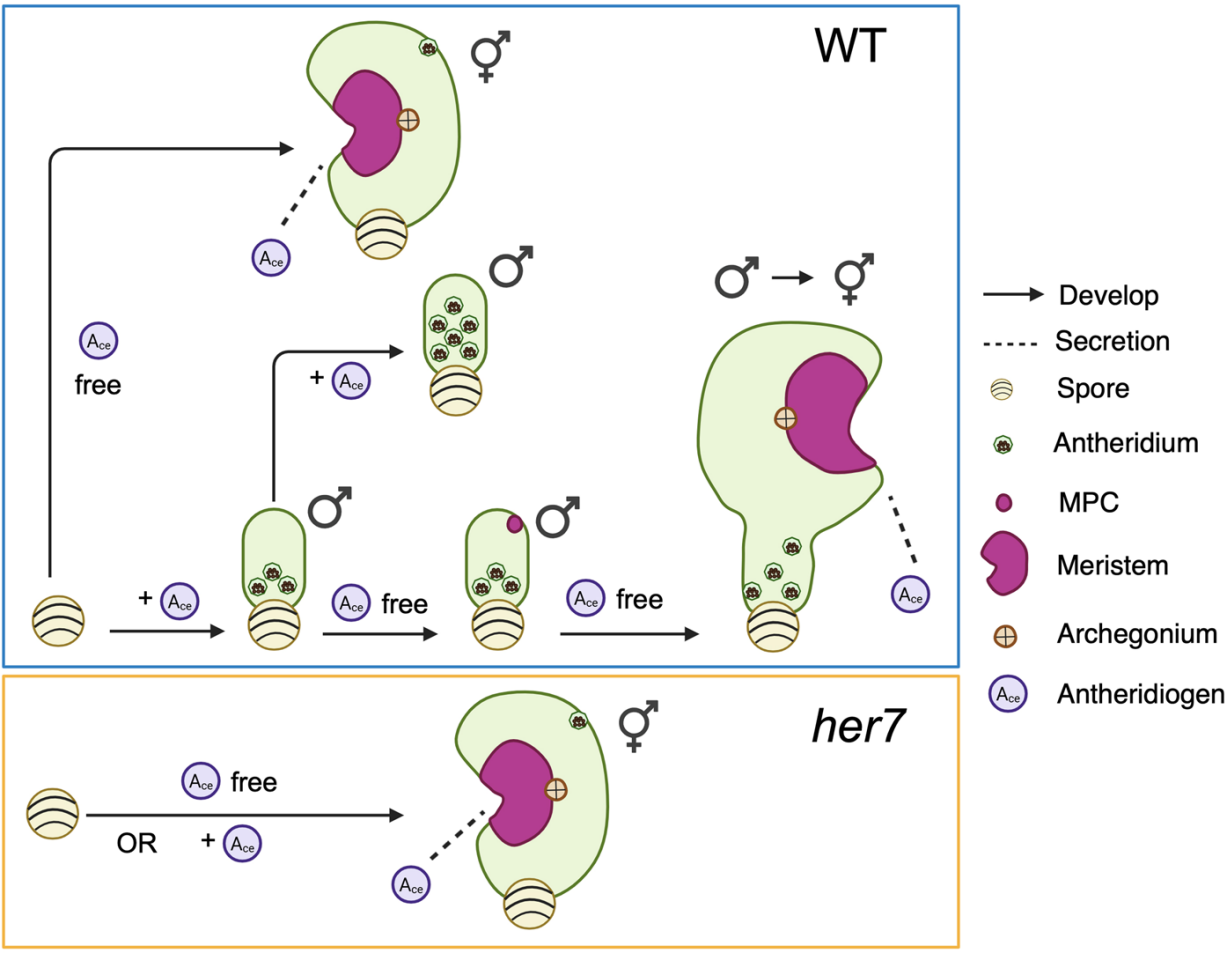
Diagram illustrating *Ceratopteris* sex determination and sex-type conversion. In WT, spores germinate and develop into hermaphrodites when not exposed to antheridiogen (A_ce_), developing a multicellular meristem and adjacent archegonia. When exposed to antheridiogen, spores instead germinate and develop into males, which lack both a meristem and archegonia but produce sperm-bearing antheridia. In the continued presence of antheridiogen, males maintain their identity and produce additional antheridia. In contrast, removal of antheridiogen triggers male-to-hermaphrodite conversion, and a meristem-progenitor cell (MPC) is established within the male body, and its progeny give rise to the entire *de novo* meristem. In *her7* mutants, spores develop into hermaphrodites in both antheridiogen-free and antheridiogen-present conditions. The figure was created using BioRender and further modified in PowerPoint for clarity

Under the influence of the pheromone antheridiogen, *Ceratopteris* gametophytes germinating from genetically identical spores exhibit sexual dimorphism, developing either as males or hermaphrodites [6, 23] (Figs. 1A-C and 2). *Ceratopteris* haploid spores that germinate in an antheridiogen-free environment, or that germinate early within a population before antheridiogen accumulates, develop as hermaphrodites (Figs. 1, 2, 3 and 4). They initiate a multicellular marginal meristem and subsequently form egg-producing archegonia adjacent to the meristem (Figs. 1A, 2 and 4). Once initiated, the hermaphrodite developmental program is generally irreversible by antheridiogen, as the marginal meristem remains indeterminate, sustains cell proliferation, and induces archegonium formation. Meanwhile, hermaphrodites can also produce a few sperm-producing antheridia outside the meristem, typically at the edges of the prothallus [6, 12, 24]. Once specified, *Ceratopteris* hermaphrodites begin producing and releasing antheridiogen into the surrounding environment [6, 23], which induces late-germinating, undetermined gametophytes within the population to develop as males. Unlike hermaphrodites, males are ameristic, lacking any meristem or archegonia, but gradually develop a number of sperm-producing antheridia (Figs. 1B, 3 and 4). Upon maturation, antheridia rupture and release motile, flagellated sperm, which swim into the archegonia of hermaphrodites to fertilize eggs and producing diploid embryos, advancing the life cycle [11, 12, 23]. Through this process, antheridiogen adjusts sex ratios in the *Ceratopteris* gametophyte population and facilitate sexual reproduction.

Antheridiogens from several ferns have been identified as gibberellin-related compounds [25–27]. For example, in *L. japonicum*, the antheridiogen shares a common ent-gibberellane skeleton with gibberellin A4 (GA4), but differs structurally by lacking an OH group at C3 and possessing a methyl-esterified carboxyl group at C6 [25, 27, 28]. In contrast, the chemical structure of the antheridiogen in *Ceratopteris richardii* remains unknown. Nevertheless, it has been proposed that Ceratopteris antheridiogen signaling likely, or at least in part, involves the GA pathway [10, 29] based on previous experimental observations. For instance, treatment of GA biosynthetic inhibitors reduced the proportion of males in *Ceratopteris* gametophyte populations, suggesting that antheridiogen and GA share similar or overlapping biosynthesis steps in *Ceratopteris* [29]. Additionally, abscisic acid (ABA) antagonizes antheridiogen actions, inhibiting antheridiogen-induced antheridium formation and male development in *Ceratopteris* gametophytes [10]. Several *Ceratopteris* mutants have been isolated that are insensitive to ABA treatment, enabling the antheridiogen-triggered male differentiation programs to proceed even in the presence of ABA [30–32]. Among them, one mutated gene has been identified as *GAIA1*, which encodes a close homolog of Arabidopsis OST1, a conserved SnRK2 signaling component [32]. Despite these advances, the cellular basis of ABA-mediated cell fate specification and cell proliferation during fern gametophyte development remains to be uncovered. Additionally, in seed plants, antagonism between gibberellin and ABA regulates various developmental progresses, including seed dormancy and germination, root growth, and response to environmental stimuli [33]. Dissecting the molecular mechanisms that mediate the antagonistic interaction between antheridiogen and ABA during Ceratopteris sex determination will be an exciting direction for future research.

Forward genetic screens have identified several mutants affecting sex type in *Ceratopteris* gametophytes, including *hermaphroditic* (*her*), *transformer* (*tra*), and *feminization* (*fem*) mutants [6, 11, 31, 34]. *her* gametophytes are insensitive to antheridiogen, and upon germination, they develop into hermaphrodites even in the presence of antheridiogen [31] (Figs. 3A-B and 4). The *tra* mutation disrupts both meristem and archegonia formation, causing gametophytes to differentiate into ameristic males even under conditions that normally promote hermaphrodite development [31]. In contrast, *feminization* (*fem*) mutants fail to form antheridia and instead promote meristic female development in gametophytes [31]. Recently, *her7* mutations were identified in a *brassinosteroid receptor-like kinase* homolog in *Ceratopteris* [35]. Bulk segregation and sequencing revealed that five independent *her7* alleles (*her7-1*, *her7-11*, *her7-14*, *her7-15*, *and her7-19*) localized to the same locus within the mapping window on chromosome 29, each carrying either missense mutations or small amino acids deletions in this receptor-like kinase homolog [35]. HER7 localizes to the plasma membrane and cytoplasm, consistent with its proposed role as a receptor kinase [35]. Transcriptomic analyses comparing wild type and *her7* mutants at 12 days post inoculation, as well as young gametophytes (4.5 days post inoculation) treated with or without antheridiogen, revealed global gene expression changes in response to antheridiogen, many of which appear to be dependent on HER7 [35, 36]. Specifically, differential expression of genes involved in brassinosteroid (BR) and GA biosynthesis and signaling provides important molecular clues for antheridiogen sensing and points to promising directions for future studies.

### Maintenance of hermaphrodite identity and meristem activity

As described above, a key regulatory mechanism ensuring the stability of this system is that, though antheridiogen induces male developmental programming, hermaphrodites, once specified, become insensitive to antheridiogen produced either by themselves or neighboring individuals. Without this insensitivity, hermaphrodites would not be able to maintain their sex type, leading to male dominance across the population. A central outstanding question in the field is: what molecular mechanism orchestrate meristem identity maintenance and antheridiogen insensitivity in hermaphrodites? Recent studies have identified a HAIRY MERISTEM (HAM) family GRAS-domain transcriptional regulator as a key player in this process [20, 37]. HAM proteins are widely present across plant lineages and are evolutionarily conserved in land plants [38, 39]. In seed plants such as Arabidopsis, the HAM family play essential roles in maintaining stem cell indeterminacy and proliferation within shoot apical meristems [39–44]. In *Ceratopteris*, the *HAM* homolog (*CrHAM*) was shown to suppress antheridiogen-mediated male differentiation while maintaining meristem indeterminacy and cell proliferation in hermaphrodites, thereby contributing to the regulation of sex ratios in gametophyte populations [20].

CrHAM protein preferentially accumulates in the meristems of hermaphrodites but is excluded from antheridia in males [20]. *CrHAM* knockdown (KD) in *Ceratopteris* transgenic lines increased the male ratio in gametophyte populations (Fig. 3C) and impaired hermaphrodite development, resulting in smaller prothalli, reduced meristem size, and a shallower meristem notch compared with wild type (WT). In many KD hermaphrodites, archegonia were absent or greatly reduced near abnormal meristems, whereas antheridia formed ectopically around and even within the meristem. Consistently, male-specific *GAMYB* genes were up-regulated in *CrHAM* KD gametophytes. In antheridiogen treatment assays, WT hermaphrodites retained their sex identity, but a considerable number of young *CrHAM* KD hermaphrodites failed to maintain meristem activity and gradually converted into ameristic males, explaining the elevated male ratio in KD populations. These results suggest that *CrHAM* is required to maintain meristems in an undifferentiated state when exposed to antheridiogen. In addition to suppressing male traits, *CrHAM* promotes female traits by sustaining cell proliferation during hermaphrodite development [20], as *CrHAM* KD exhibited reduced prothallus size and cell number due to decreased division activity. Transcriptomic analyses further reflected the positive roles of *CrHAM* in promoting meristem activity and hermaphrodite development. Gene Ontology (GO) analysis of differentially expressed genes (DEGs) in *CrHAM* KD hermaphrodites indicate *CrHAM* involvement in cell growth and prothallus expansion [20]. Additionally, a *CLAVATA1* homolog (*CrCLV1a*) and an auxin biosynthesis gene, the *YUCCA* homolog (*CrYUC1*) were downregulated in *CrHAM* KD hermaphrodites. Both genes are generally expressed at higher levels in WT hermaphrodites compared with WT males [20]. This finding was consistent with studies in the liverwort *Marchantia*, where *MpCLV1* and *MpYUC2* are expressed in the meristem and required for cell proliferation in the notch [45, 46].

The transcriptional reporter showed that the *CrHAM* promoter is active in nearly all cells of hermaphrodite prothalli (except gametes), whereas the translational reporter revealed CrHAM protein enrichment specifically at the meristem [20] (Fig. 3D). This raises an important question: what factors control the spatial restriction of CrHAM protein accumulation? In Arabidopsis, type II *HAM* genes (*AtHAM1*, *AtHAM2* and *AtHAM3*) are negatively regulated by microRNAR171(miR171) [38, 39, 47, 48]. In Arabidopsis SAMs, *MIR171/170* are specifically expressed in the epidermis and move downwards within a limited distance into deeper layers, and they mediate the cleavage of *AtHAM1-3* transcripts, generating an apical–basal concentration gradient of HAM proteins [49, 50]. Interestingly, the *CrHAM* coding sequence contains a miR171-binding site (5’-GATATTGGCGCGGCTCAATCA-3’) identical to that in *AtHAM1-3* genes, indicating a conserved regulatory mechanism [39]. Supporting this, two *Ceratopteris MIR171 genes* (*CrMIR171B* and *CrMIR171C*) have been identified, and their mature miRNAs share nearly identical sequences with Arabidopsis miR171b/c [20]. Overexpression of *CrMIR171B* in *Ceratopteris* reduced *CrHAM* expression and produced defects resembling *CrHAM* KD, including an increased male ratio in gametophyte populations (Fig. 3C, D), and smaller prothalli and shallower meristem notches in hermaphrodites [20]. Together, these findings provide evidence that the miR171 family is present and functional in *Ceratopteris*, where it contributes to sex determination and meristem maintenance likely through negatively regulating *CrHAM* expression [20, 37, 39]. This highlights a conserved yet lineage-specific miR171-*HAM* regulatory module in ferns, opening promising directions for future studies.

### Male-to-hermaphrodite conversion and *de novo* meristem formation

*Ceratopteris* gametophytes exhibit remarkable sexual developmental plasticity, with sex types capable of switching in response to environmental cues (Fig. 4). Previous studies demonstrate that *Ceratopteris* hermaphrodites continuously produce and secret antheridiogen, which not only induces sexually undetermined gametophytes to develop as males but is also required for maintaining the male developmental program [12]. When antheridiogen is removed or depleted, male identity cannot be maintained, and male gametophytes initiate *de novo* meristems and produce egg-bearing archegonia, thereby converting into hermaphrodites [12, 22, 23](Figs. 1C and 4). This environmentally responsive strategy allows ferns to adjust population sex ratios under changing conditions, promoting outcrossing and enhancing reproductive success. This naturally occurring male-to-hermaphrodite conversion provides an ideal model for dissecting the molecular and cellular mechanisms of cell fate specification and the coordination of cell division during *de novo* meristem formation. Recent work has combined long-term, non-invasive time-lapse confocal imaging with computational image analysis of a ubiquitously expressed fluorescence nuclear marker, capturing the complete developmental trajectory of this process at high spatiotemporal resolution [22].

This study reconstructed the dynamic lineage maps of sex-type conversion and revealed that the entire newly formed multicellular meristem originates from a single non-antheridium cell, the meristem progenitor cell (MPC), in the male prothallus [22] (Fig. 4). Over time, the MPC lineage expands and sustains active cell division, in contrast to other cell lineages, which gradually become mitotically inactive during meristem formation and sex-type conversion. In parallel, a mathematical model demonstrated that a combination of stochastic cell division and inhibitory signals from actively dividing cells is sufficient to account for MPC lineage establishment and meristem formation [22]. Furthermore, treatment with Aphidicolin, a drug that specifically inhibits the S phase of the cell cycle, disrupted male-to-hermaphrodite conversion and led to the reduced size of *de novo* meristems [22], consistent with the simulation generated using adjusted parameters for reduced cell division. These findings demonstrated that re-entry into and maintenance of cell cycle progression are essential for male-to-hermaphrodite conversion and MPC lineage establishment [22].

Division orientation and cell position further influence division activity during *de novo* meristem formation. In particular, the orientation of the initial division in the MPC determines the proliferative activity of its progeny: an anticlinal division produces two daughter cells in the outermost layer, giving rise to two lineages with similarly high division activity, whereas a periclinal division generates one daughter cell in the outermost layer, which maintains higher division activity in its lineage, and another in the inner layer, which produces a cell population with lower activity [22]. Quantitative analyses show that, once *de novo* meristems are established, cells in the outermost layer divide significantly more frequently than those in the inner layers [22]. This pattern is consistent with previous findings that the outermost (marginal) layer of meristem cells in *Ceratopteris* hermaphrodites exhibits more active division activity compared with inner cells [17]. Interestingly, recent work in the epiphytic fern *Phlebodium pseudoaureum* (Polypodiaceae) also demonstrated that the outermost layer predominantly drives active cell division during *de novo* meristem formation and notch establishment [51]. Together, these results suggest that in *Ceratopteris* gametophytes, meristem development during normal hermaphrodite growth and *de novo* meristem formation during sex-type conversion share conserved strategies, driven by positional signals from the outermost layer, to sustain cell proliferation. These strategies also appear to be conserved across fern species from diverse taxa.

## Future perspectives

Over the past few years, significant progress has been made in uncovering the molecular and cellular mechanisms underlying sex determination in fern gametophytes. Building on both classical and recent studies, multiple hormonal and genetic factors have been identified, yet important new questions continue to emerge. Hormones such as gibberellins, ABA, and BR have all been implicated in *Ceratopteris* sex determination [10, 26, 27, 29, 32, 35]. Future efforts to dissect the complex hormonal crosstalk that governs sexual differentiation in *Ceratopteris* gametophytes will be particularly informative. For instance, comparative transcriptomic profiling suggests dynamic regulation of BR and GA biosynthetic pathways during *Ceratopteris* sex determination [35]. At 12 days (d) after inoculation, hermaphrodites show higher transcript levels of BR biosynthetic genes, including two *CYP90B* homologs and one *CYP90C* homolog, compared with males [35]. In contrast, 4.5 d gametophytes treated with antheridiogen show reduced expression of a few GA biosynthetic genes, including one *ENT-COPALYL DIPHOSPHATE SYNTHETASE/ENT-KAURENE SYNTHASE* (*CPS*/*KS*) and one *GA20OX* homolog [35]. It will be important to examine how both BR and GA biosynthesis contribute to *Ceratopteris* sex determination. Additionally, antheridiogen treatment upregulates several ABA signaling genes, suggesting another layer of negative regulation [36]. It will be crucial to dissect the antagonism between antheridiogen and ABA, at cellular and molecular levels, in regulating sex-type specification after spore germination, during meristem development in hermaphrodites, and throughout sex-type conversion. Moreover, recent work suggested a miR171-HAM regulatory module involving in *Ceratopteris* sex-determination and meristem development [20, 37]. Future studies could focus on dissecting the function and regulation of the *Ceratopteris MIR171* family, including identifying additional members, mapping their dynamic expression patterns, defining upstream regulators, and distinguishing overlapping versus distinct functions of individual *CrMIR171* genes. Addressing these questions will benefit from recently developed tools and resources in *Ceratopteris*, including stable transformation and reverse genetics [52–54], forward genetics [35], non-invasive quantitative live imaging [17, 24, 55–57], deep learning-based image analysis [58], computational modeling [22], and multi-omics approaches [19, 20, 59, 60]. Together, these directions have strong potential to provide deeper molecular insights into the interacting regulatory networks governing fern sex determination and meristem activity.

## Data Availability

No datasets were generated or analysed during the current study.
